# Biorheology of occlusive thrombi formation under high shear: *in vitro* growth and shrinkage

**DOI:** 10.1038/s41598-020-74518-7

**Published:** 2020-10-29

**Authors:** Britt J. M. van Rooij, Gábor Závodszky, Alfons G. Hoekstra, David N. Ku

**Affiliations:** 1 Computational Science Lab, Institute for Informatics, University of Amsterdam, Amsterdam, The Netherlands; 2 GWW School of Mechanical Engineering, Georgia Institute of Technology, Atlanta, USA

**Keywords:** Biomedical engineering, Cardiovascular diseases

## Abstract

Occlusive thrombi formed under high flow shear rates develop very rapidly in arteries and may lead to myocardial infarction or stroke. Rapid platelet accumulation (RPA) and occlusion of platelet-rich thrombi and clot shrinkage have been studied after flow arrest. However, the influence of margination and shear rate on occlusive clot formation is not fully understood yet. In this study, the influence of flow on the growth and shrinkage of a clot is investigated. Whole blood (WB) and platelet-rich plasma (PRP) were perfused at high shear rates (> 3,000 s^−1^) through two microfluidic systems with a stenotic section under constant pressure. The stenotic section of the two devices are different in stenotic length (1,000 vs 150 μm) and contraction angle of the stenosis (15° vs 80°). In all experiments, the flow chamber occluded in the stenotic section. Besides a significantly increased lag time and decreased RPA rate for PRP compared to WB (*p* < 0.01), the device with a shorter stenotic section and steeper contraction angle showed a shear-dependent occlusion and lag time for both PRP and WB. This shear-dependent behavior of the platelet aggregate formation might be caused by the stenotic geometry.

## Introduction

High shear thrombosis can cause myocardial infarction or ischemic stroke by the rapid formation of an occlusive thrombus on a ruptured atherosclerotic plaque. This happens at pathological shear rates (> 4,000 s^−1^). The von Willebrand factor (vWF) together with platelets are a key factor for this process which consists of platelet adhesion and aggregation^1^. The vWF is dependent on the shear rate and has a globular shape at low shear rates (< 1,000 s^−1^) and elongates at high shear rates. The elongation threshold is around 1,000 s^−1^ when a vWF is attached to the wall and around 5,000 s^−1^ for a free-flowing vWF^2^. A balance is present between growth and shrinkage of a thrombus via platelet accumulation, embolization, and contraction^3^. It is still unknown what makes this balance shift to growth during thrombosis. Microfluidic devices are tools that have been frequently used to investigate mechanical and biochemical mechanisms of thrombus formation^4–8^. The role of the hemodynamics on the clot formation is not fully understood yet, i.e. the influence of shear rate, platelet margination and the cell-free layer. From cell-based blood flow simulations, it has been observed that the platelet flux increases due to the presence of red blood cells (RBCs). In addition, the residence time of platelets is lower close to the wall and the local shear rate is influenced by the presence of RBCs [Van Rooij et al., submitted for publication]. The objective of this study is to investigate the influence of the shear rate, platelet margination and the hemodynamics on platelet aggregate growth and shrinkage using two microfluidic flow chamber with a different geometry.

The growth of an occlusive thrombus is investigated using the three phases in which high shear thrombus growth can be divided: (1) lag phase, (2) rapid platelet accumulation (RPA) growth, (3) occlusion^9^. The lag time is thought to be the time it takes for vWF to adhere to the thrombogenic surface and to bind the first couple of platelets. Those platelets can become activated by high shear rate due to high tensile forces on the GPIb-vWF bond or biochemical agonists^10–12^. Next, the RPA phase starts, in which the platelet aggregate grows rapidly until occlusion of the vessel is reached. These phases have been studied with different shear rates and concentrations of chemicals. Bark et al.^13^ found that the lag time decreases with increasing shear rates in stenotic glass tubes. Additionally, they reported the highest thrombus growth rate at a shear rate of approximately 6,000 s^−1^. Clot growth under different shear rates in a microfluidic device has been investigated by Li et al.^14^. They reported a decrease in occlusion time with increasing shear rate (500–10,000 s^−1^). The same relation was found for the lag time which agrees with the results of Bark et al.^13^. Casa et al.^15^ investigated the influence of pulsatility on the lag time and rapid platelet aggregation for ${\dot{\gamma }}=3,800 {-} 16,000\hbox { s}^{-1}$ and found no significant difference. Ruggeri et al.^16^ found that soluble vWF is necessary for the formation of platelet aggregates at high shear rates (24,000 s^−1^). This was also observed by Para et al.^17^, who showed that soluble vWF gave more thrombus growth than surface-immobilized vWF. In addition, Casa et al.^18^ investigated the influence of the vWF and platelets at various concentrations. They reported that vWF is the primary mediator of high shear thrombosis and hypothesized that vWF stored in α-granules of platelets may be critical for occlusive thrombus formation. This implies that platelet activation is needed to start the RPA phase. This activation is dependent on the shear-induced interaction between GPIb and vWF and is called shear-induced platelet aggregation (SIPA)^19^. According to literature, platelet activation after high shear stress is often observed after about one minute upon release of the α-granules^20^. For thrombin mediated activation, α-granules are released in about 30 s^21^.

The shrinkage of an occlusive thrombus has been investigated in previous work, which is defined in this study as the normalized area of shrinkage per time. The mechanism of clot shrinkage caused by platelets has been studied less for healthy and diseased blood. Contraction of a platelet is caused by the interaction of non-muscular myosin II, present in the cytosol of a platelet, and actin filaments of the platelet cytoskeleton. The myosin and actin are connected to fibrinogen or vWF via the integrin α_IIb_β_3_ that is located in the membrane of a platelet^22^. The contractile forces of the platelet cytoskeleton are transferred to the extracellular matrix which results in clot contraction. Ono et al.^23^ demonstrated that clot contraction occurs under flow (${\dot{\gamma }}=1,800\hbox { s}^{-1}$) during the primary hemostasis and showed that it is independent of thrombin and fibrin. This contraction was found to be most rapidly at the downstream side of the developing thrombus. The contractile mechanism is dependent on signaling by Rho kinase. In addition, they stated that the contraction makes the aggregate more stable. Auger et al.^24^ showed fibrin-independent contraction of platelet aggregates against the direction of flow at the front of the platelet aggregate (${\dot{\gamma }}=1,000\hbox { s}^{-1}$). They found that the contraction is independent of ADP and thromboxane. It is a dynamic process which involves Src kinases and actin polymerization. Muthard and Diamond^7^ studied the effect of flow arrest on the contraction of clots that have been formed under high shear rates (1,160 s^−1^). The abrupt stop of flow increased the clot contraction rate by 5–6×. The contraction was dependent on ADP and thromboxane that increased in concentration when the flow was stopped. Therefore, this clot contraction was mediated by platelet contraction, however, triggered in a different way compared to^24^ and^7^. Muthard and Diamond^7^ found a higher contraction rate downstream of the clot which was also observed by^23^ and^24^. Chen et al.^25^ investigated the contraction of micro-clots in a dynamic flow environment and reported an equal influence of shear flow and biochemical substances on micro-clot contraction. Additionally, they found a higher clot contractile force and a higher platelet density for higher shear rates (3–1,500 s^−1^).

These mechanisms will influence the location, size and porosity of the clot. This knowledge could help to find new treatments to thrombosis or new methods to prevent it from happening. In this study, we investigate the formation of occlusive thrombi in two different flow environments caused by a different shaped stenotic section. Two microfluidic devices were used in this study: an existing flow chamber developed by Casa et al.^18^ that contains a gradual stenosis and a newly designed flow chamber, called Van Rooij’s flow chamber, which contains a sudden stenosis. The pathophysiological relevance of Casa’s flow chamber might be an atherosclerotic plaque and Van Rooij’s flow chamber might mimic a micro-medical device. A higher shear gradient is expected at the corners of the sudden stenosis which might lead to formation of a clot at the downstream contraction side, as was shown by Tovar-Lopez et al.^26^. In addition, the influence of platelet margination on the three phases in thrombus formation is investigated. In order to do so, experiments with whole blood (WB) and platelet-rich plasma (PRP) were performed and occlusion times, lag times and rapid platelet accumulation rates were obtained. To the best knowledge of the authors, this is the first time that shrinkage of an occlusive thrombus formed under pathological high shear rates (> 6,000 s^−1^) in PRP is observed in two differnt flow chambers. Furthermore, we report the observation of platelet aggregates that have a shape similar to mountains and valleys, in PRP.

## Results

### Occlusion times and Lag times

**Figure 1:**
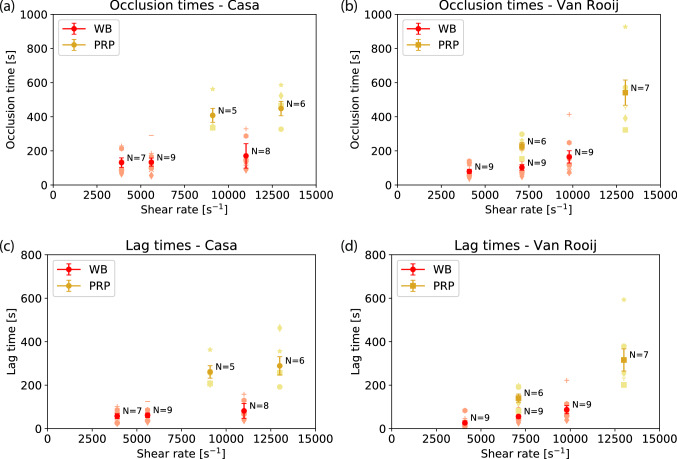
The average occlusion times and standard error for each pressure difference are shown for the PRP and the WB experiments using (**a**) Casa’s and (**b**) Van Rooij’s flow chamber. In (**c**) and (**d**) the average lag times are shown for Casa’s and Van Rooij’s flow chamber, respectively. The number of blood samples N are given for each type of experiment. Each symbol represents the average of at least two experiments performed with the same blood sample.

For Casa’s flow chamber, no relation is found between the occlusion times and the shear rate (see Fig. 1). The same holds for the lag times for Casa’s flow chamber. For Van Rooij’s flow chamber significantly longer occlusion times (t = 4.13, df = 16) and lag times (t = 4.77, df = 16) have been found between the lowest shear rate (4,100 s^−1^) and the highest shear rate (9,800 s^−1^) (*p* < 0.01). In addition, a significantly increased occlusion time of about 2.2 – 2.5× for the Casa chip (t = 8.55, df = 12) and 2.2 – 3.2× for the Van Rooij chip (t = 5.03, df = 13) was found for the PRP experiments compared to the WB experiments at approximately 6,000 s^−1^ (*p* < 0.01). The occlusion time at the highest shear rate case in Van Rooij’s chip for PRP (13,000 s^−1^) seems to increase more (3.2×). Moreover, the lag time is significantly increased by 3.2× for PRP in Casa’s chip (t = 6.12, df = 11) and by 2.5 – 3.0× for PRP in Van Rooij’s chip (t = 3.83, df = 13) compared to WB (*p* < 0.01).

### Rapid platelet accumulation rate

**Figure 2:**
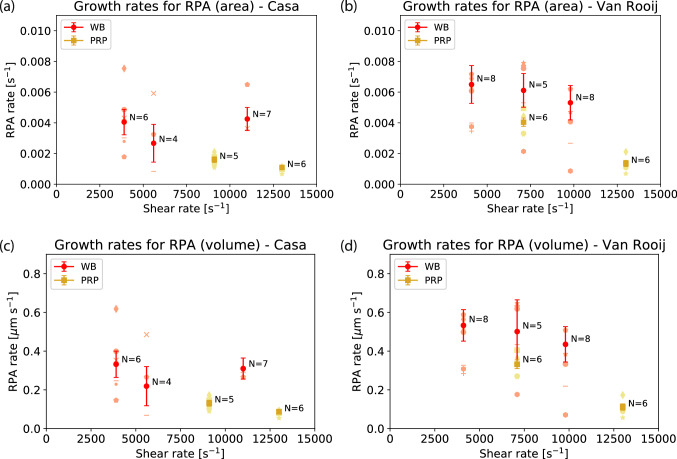
The average rapid platelet aggregation (RPA) rate and standard error in normalized area per second are shown for the PRP and the WB experiments using (**a**) Casa’s and (**b**) Van Rooij’s flow chamber. In (**c**) and (**d**) the averaged RPA rate in volume per second is given for Casa’s and Van Rooij’s flow chamber, respectively. The number of blood samples N are given for each type of experiment. Each symbol represents the average of at least two experiments performed with the same blood sample.

In Casa’s flow chamber, the RPA rate does not seem to be a strong function of shear rate for either for WB (3,900–11,000 s^−1^) and PRP (9,100–13,000 s^−1^) (see Fig. 2a). For Van Rooij’s flow chamber, this is also true for WB (4,100–9,800 s^−1^); however, for PRP the RPA rate is significantly lower (t = 7.44, df = 10) at a higher shear rate (7,100–13,000 s^−1^) (*p* < 0.01) (see Fig. 2b). In addition, in Casa’s (t = 9.09, df = 7) and Van Rooij’s (t = 3.58, df = 9) flow chamber the RPA rate is significantly lower in PRP compared to WB (*p* < 0.01). This was found when comparing the RPA rate in WB at 9,100 and 11,000 s^−1^ with the RPA rate in PRP at 13,000 s^−1^ for Casa’s flow chamber (see Fig. 2a), and comparing the RPA rate among PRP and WB at 7,100 s^−1^ (see Fig. 2b) for Van Rooij’s flow chamber. For shear rates < 10,000 s^−1^ in PRP, we found a significantly lower RPA rate for Casa’s flow chamber in comparison to Van Rooij’s flow chamber (*p* < 0.01). When the growth in the y-direction (see Fig. 6) was incorporated, a similar behavior is observed (see Fig. 2c-d).

### Shrinkage rate

**Figure 3:**
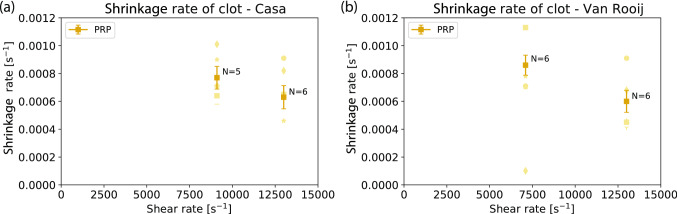
The average shrinkage rate and standard error are shown for the PRP experiments for (**a**) Casa’s chip and (**b**) Van Rooij’s flow chamber for the normalized area. The number of blood samples N are given for each type of experiment. Each symbol represents the average of at least two experiments performed with the same blood sample.

The shrinkage rates are measured as normalized area change per unit time in PRP (see Fig. 3). It was not possible to measure this rate in WB due to stagnation of blood. This stagnation increased the amount of light that hits the sensor of the camera (see Supplementary Information video S1 or S3 online). For both flow chambers, the shear rate and geometry did not influence the shrinkage rate. The average shrinkage rate in PRP was found to be 7.1 ± 0.35·10^−4^ s^−1^.

### Mountains-and-valleys-like shaped aggregates at high shear rates

**Figure 4:**
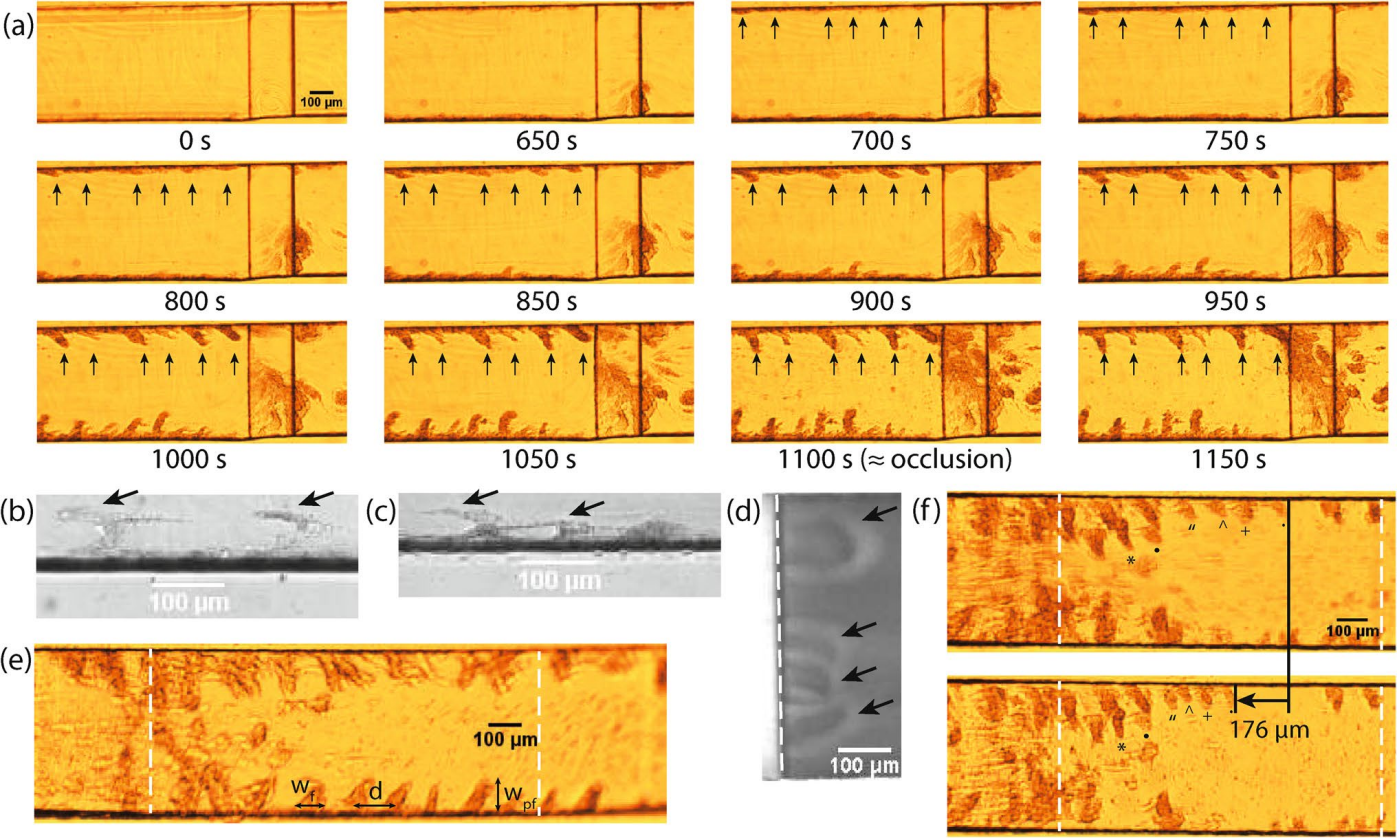
The mountains-and-valleys-like shaped aggregates and U-shaped strings are shown. The start and/or end of the stenotic section is assigned by dashed white lines. The flow-direction in all images is from left to right. (**a**) The formation of mountains-and-valleys-like shaped aggregates from the side in Van Rooij’s flow chamber at different time points. The black arrows point at the forming mountain-like shaped aggregates. (**b**)–(**c**) Examples of U-shaped strings in Van Rooij’s chip at the corner of the channel in PRP experiments (green channel of microscopic image). The black arrows point to the strings. (**d**) U-shaped strings (see black arrows) attached to the downstream corner of the stenotic section of Van Rooij’s flow chamber in a WB experiment (red channel of microscopic image). (**e**) The mountains-and-valleys-like shape is measured as shown in this figure, where w_f_ is the width of a mountain in the flow direction measured at the base of the mountain, d is the distance between the middle of two mountains bases, w_pf_ is the width at the mountain perpendicular to the flow direction. (f) The shrinkage of a long fibre with platelet aggregates, each platelet aggregate is marked with a different symbol.

In PRP, the formation of platelet aggregates with a mountains-and-valleys-like shape was observed at the corners of the microfluidic flow chamber during some experiments in both flow chambers. The final shape of the platelet aggregates looks like mountains that are separated by valleys with no or little platelet aggregation. The platelet aggregates that form the mountains started to grow in the direction of flow, however, when the flow starts to slow down, they raise up into the channel and seem to contract a bit to give them their final mountains-like shape, which are directed perpendicular to the flow. This process is visualized in Fig. 4a, the black arrows point at the forming aggregates. The start of the growth of these mountains seems to happen on U-shaped strings at the corners of the flow chamber (see Fig. 4b–c, green channel of microscopic image). Those strings are also visible at the downstream corner of the stenotic section in Van Rooij’s flow chamber in WB (see Fig. 4d, red channel of microscopic image), which suggest that they play a general role in platelet aggregation.

In Casa’s chip, the mountains-and-valleys-like shaped aggregates formed in the stenotic section (9,100–13,000 s^−1^, see Fig. 4e). In Van Rooij’s chip, they formed up- or downstream of the stenotic section of the flow chamber (3,000 s^−1^) as shown in Fig. 4a.

We measured the distance d between the mountains in 16 different cases and found an average of 152 ± 8 μm (see Fig. 4e). The mean of the width perpendicular to the flow w_pf_ and the mean of the width in the direction of flow w_f_ are 98 ± 8 μm and 65 ± 4 μm, respectively. In Fig. 4e it is shown how the distance and widths of the mountains-and-valleys-like shaped aggregates were measured.

### Clot shrinkage

**Figure 5:**
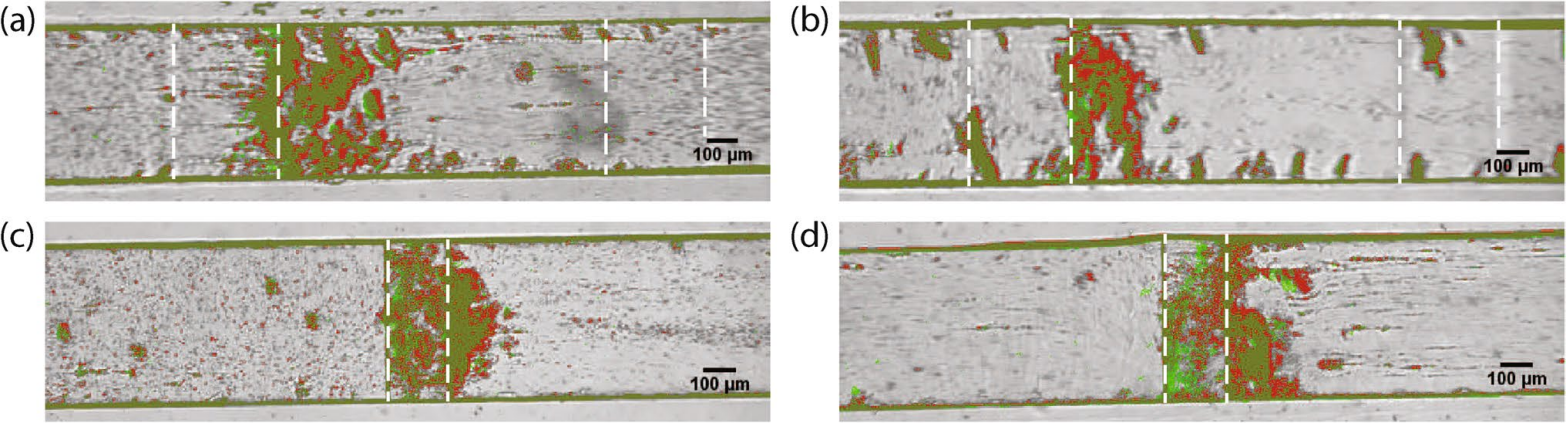
Two examples of the shrinkage of the occlusive platelet aggregate are shown for each flow chamber. The flow-direction in all images is from left to right. (**a**)–(**b**) The shrinkage in Casa’s flow chamber is shown with the stenotic section assigned with the second and third dashed white lines. (**c**)–(**d**) The shrinkage in Van Rooij’s flow chamber is shown with the white dashed line assigning the stenotic section. The color fill of the clot at occlusion time is represented in red and the color fill of the clot at the end of the experiment is shown in green. The two color fills overlap and this results in the the dark green color. The time between the two red and green fill Δt are 107 s, 124 s, 126 s and 117 s, respectively for (**a**)–(**d**).

Shrinkage of the platelet aggregates was observed during the whole experiment; however, not easy to quantify (see Supplementary Information video S2 and S4 online). When the clot occluded the flow chamber, the clot shrinkage became more visible. In Fig. 5 the size of the platelet aggregates at two time points is overlapped, one at occlusion time (red fill) and one at the time when the experiment was stopped (green fill). The part of the clot that has contracted is visible in red. The red color is more pronounced on the downstream side of the platelet aggregate, which suggests more shrinkage on the downstream than on the upstream side. In addition, some small green areas in Fig. 5 are visible which suggests that the clot moved a bit in the upstream direction due to shrinkage. For these four cases, the velocity of the shrinkage in the direction of flow is measured from the red areas in Fig. 5. We found a shrinkage velocity of 4–8 μm/min for those cases. In some experiments, we observed aggregates that seem to be connected via long strings of fibres. This network of aggregates shrinks and the aggregates move upstream closer to the stenosis as shown in Fig. 4f.

## Discussion

We report high shear rate thrombus formation in Casa’s and Van Rooij’s flow chamber. Both chips create different flow conditions: Casa’s flow chamber represents a flow environment with the presence of an atherosclerotic plaque and Van Rooij’s flow chamber represents a more sudden change in channel width and thus in flow. Our study demonstrates higher occlusion times and lag times and lower rapid platelet accumulation for the PRP experiments in comparison to the WB experiments in both flow chambers (*p* < 0.01). The shrinkage of the platelet aggregates starts during the experiments and is clearly visible in the PRP experiments when the flow stops. The shrinkage rate was similar for both flow chambers (*p* < 0.01), which suggests that the shrinkage is independent of flow condition under which the aggregate has formed. The shrinkage of the clot lowers its permeability and is therefore important to study with regarding to new therapeutic treatments.

Bark et al.^27^ reported a decrease in lag time between 0 and 6,000 s^−1^ for experiments with porcine WB in stenotic glass tubes; however, from 2,000 s^−1^ the lag time was nearly constant. Li et al.^28^ reported for human blood a decrease in lag time between 500 and 10,000 s^−1^. The decrease was most significant between 1,500 and 4,000 s^−1^. The lag times found in WB for Casa’s flow chamber indicate that it is independent of the shear rate over a range of 3,900 s^−1^ to 13,000 s^−1^). This was not the case for the Van Rooij’s chip, the lag time increased significantly between 4,100 and 9,800 s^−1^. The geometric design of the chip could explain this, a sudden change in flow rate is found at the corners of the stenotic section (see Supplementary Information Fig. S2 online).

To start the rapid platelet accumulation phase, the platelets that have been bound during the lag phase should have been activated to create an increase in vWF concentration close to the wall^29^. Casa et al.^15^ found an average lag time of 48 ± 10 s in human WB using a threshold on the volume obtained from confocal images. We found a lag time in WB in the range of 58–82 s for Casa’s chip and 26–87 s for Van Rooij’s chip. These lag times are comparable to the lag times found by Casa et al.^15^. However, for Van Rooij’s flow chamber a larger range of lag times was obtained. This could be due to the difference in geometry of the channel. The lag times that were found are long enough to activate platelets which can secrete their granules to increase the vWF concentration (< 1 minute)^20^.

The lag times and occlusion times in PRP were increased by a factor of three in both chips. Kobayashi et al.^30^ observed higher occlusion times (2.4×) in PRP compared to WB in Casa’s flow chamber. An increase in lag time (∼ 4–5×) in stenotic tubes perfused with PRP compared to WB has been reported by Mehrabadi et al.^31^. Additionally, Casa et al.^15^ showed that the plasma vWF concentration and platelet concentration are important in the lag phase. In our previous study, an increase in platelet flux of about two times in WB was found in comparison to PRP in Van Rooij’s flow chamber [van Rooij et al., submitted]. This increase is caused by enhanced diffusion due to the presence of red blood cells^32^. However, in PRP this enhanced diffusion is not present. Therefore, the concentration of platelets will be lower at the walls of the channel for PRP compared to WB. To conclude, platelet margination can be a reason for the higher lag times in PRP in comparison to WB. In addition, platelet margination might cause the significantly lower RPA rate for PRP compared to WB (*p* < 0.01). The increased occlusion time is caused by both an increased lag time and a decreased RPA rate.

In Van Rooij’s flow chamber the RPA in PRP is significantly different between a shear rate of 7,100 s^−1^ and 13,000 s^−1^. A reason for this may be that platelets move faster through the flow chamber and have less time to bind. Bark et al.^33^ reported that above a shear rate of 7,000 s^−1^ the growth of a clot is limited primarily by the kinetic binding rate of platelets. In our PRP experiments, all experiments are performed above 6,000 s^−1^. This suggests that the RPA rate in PRP will be limited by the kinetic binding rate of platelets. The residence time of a platelet should than be close to the binding time between vWF and GPIb (< 10 μs). If we assume that our cell-based PRP simulations [Van Rooij et al., submitted] represent what happens in Van Rooij’s flow chamber, a large plasma layer without platelets was found close to the wall (≈ 10 μm) for both cases and a lower residence time for 13,000 s^−1^ (≈ 3–10 μs) was found compared to 7,100 s^−1^ (≈ 10–30 μs) (see Supplementary Information Fig. S3 online). This may indicate that the kinetic binding limit of a PLT to vWF is close to this 3–10 μs. A more precise value could be found by performing PRP experiments at higher shear rates (> 13,000 s^−1^) and check at which shear rates no aggregates could be formed.

In addition, the RPA rate in WB seems to be lower in Casa’s flow chamber in comparison to Van Rooij’s flow chamber over a shear rate range of 3,900–11,000 s^−1^. Therefore, we assume that the stenotic geometry of Van Rooij’s flow chamber is responsible for the higher RPA rate found in this flow chamber compared with Casa’s flow chamber. In continuum and cell-based blood flow simulations [Van Rooij et al., submitted] it was found that high shear rate gradients are present at the corners of the stenotic section, due to the steep contraction and expansion angle (see Fig. S2). This high shear rate gradient might influence the RPA rate. However, high shear rate gradients seem not to be necessary to form platelet aggregates under high shear rate, since platelet aggregation was also found in Casa’s flow chamber.

The RPA rate in volume growth per second, normalized for the surface area, was also measured. In order to do so, the whole stenotic area was taken into account. Note that generally the corners of the flow chamber are excluded from the analysis due a non-uniform shear. Since, the devices have different flow conditions at the edges of the stenotic sides, due to a different contraction angle, it was decided to include the corners.

Contraction of the clot is known to be a process that is actomyosin-based and dependent on Ca^2+^-ions. In our experiments the observed clot shrinkage might be a combination of this actomyosin contraction and a reduction in drag force upon flow arrest. This reduction of drag force could result in an elastic spring effect which could be a material behavior of the vWF. From histology analysis (Carstairs staining) of previous experiments in capillary tubes performed by Mehrabadi et al.^34^, it was found that the clots mostly contain platelets for clots formed in WB and PRP. Therefore, it is possible that shrinkage of the occlusive clots in our experiments can be influenced by a combination in actomyosin contraction and reduction in drag force. In the future, experiments with the use of an actin-blocker or myosin-blocker could be performed to find the main contributor to this process. In addition, it would be interesting to know if the actomyosin-based contraction is triggered by biochemical agonist, such as ADP and thromboxane, or by Rho kinase signaling. The shrinkage rate measured in PRP was comparable between both flow chambers and it, therefore, is assumed that it is independent on the flow rate. The averaged value of all experiments was 7.1 ± 0.35·10^−4^ s^−1^ defined over a time interval of ≈ 60–120 s. To get more insight in this flow independence and the duration of the shrinkage, further research is needed. Muthard and Diamond et al.^7^ reported an average clot contraction velocity of 4.5 μm/min after one minute that was measured with fluorescent beads. The contraction velocity found by^7^ was much higher downstream (≈ 6 μm/min) than upstream (≈ 3 μm/min). From the microscopic images we also observed more shrinkage downstream as is shown in Fig. 5. For four experiments (shown in Fig. 5) the shrinkage velocity was measured and was found to be around 4–8 μm/min on the downstream side of the clot. Hence, these values are of the same order of magnitude as the contraction velocity found by^7^.

Mountains-and-valleys-like shaped aggregates were observed in both flow chambers in PRP experiments. This phenomenon has been observed in capillary tubes as well by Casa et al.^15^. However, the physical mechanism behind it stays unclear. The mountains-and-valleys-like shaped aggregates might be connected to the way vWF networks form on the collagenated surface, i.e. U-shaped vWF. In our experiments, U-shaped strings orientated perpendicular to the flow, that seem to catch many platelets, were observed (see Fig. 4c–e). These U-shaped strings were observed by Colace et al.^10^ as well. We assume that the strings are vWF, however, further investigation is needed to clarify this. Furthermore, the formation seems to have a regular pattern. Therefore, the average distance between mountains and their width in flow direction and perpendicular to the flow direction was measured and presented. This regular pattern might be related to the length of the von Willebrand factor that plays a role in the aggregation; however, further research is needed to confirm this. Another reason for the formation of these shapes at the sides of the channel might be a higher concentration of collagen fibres at the corners of the microfluidic channel. The quantification of those mountain-and-valley-like shapes has not been reported before, as far as the authors know.

## Materials and methods

### Whole blood and Platelet-rich plasma

Whole porcine blood obtained from a slaughterhouse (Holifield Farms, Covington, USA) was anticoagulated with heparin (3.5 USP units/mL) directly after electrical stunning of the pigs. The platelet-rich plasma was prepared by sedimentation of the red blood cells. After 2 h the PRP layer was transferred in 50 mL tubes using a pipette. The experiments were performed within 6 h, starting from the moment that the blood was collected from the pigs.

### Microfluidic chip designs

**Figure 6:**
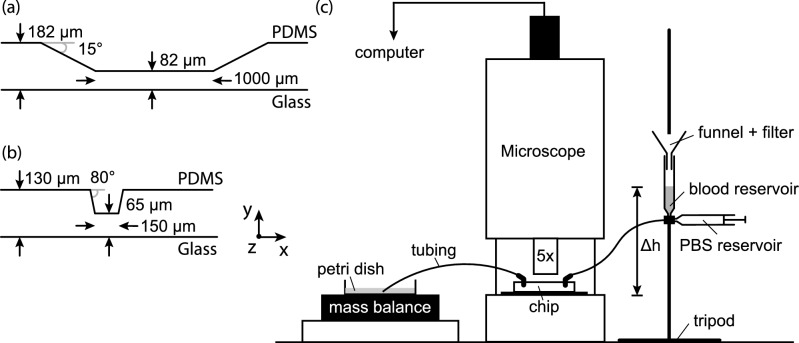
The experimental set-up including the used microfluidic devices are shown. Side view of the geometric design of (**a**) Casa’s flow chamber and (**b**) Van Rooij’s flow chamber. The depth of both flow chambers is 480 μm (z-direction, not shown). (**c**) A schematic representation of the experimental set-up. These figures are reused from [Van Rooij et al., submitted for publication].

Two microfluidic devices were used in this study. One device that contains a gradual stenosis has been designed by Casa et al.^5,35^ and used in previous studies^5,36^ (see Fig. 6a). The second device was newly designed and was introduced in a previous study by [Van Rooij et al., submitted for publication]. This new flow chamber contains a stenotic section with a steeper contraction and expansion area of 80° and a shorter stenotic length of 150 μm than Casa’s flow chamber (see Fig. 6b). The design of the stenotic section is based on the flow chamber used by Tovar-Lopez et al.^26^: 80° contraction angle and 20 μm stenotic length. The influence of the steeper contraction and expansion angle of the stenosis and the length of the stenotic section on thrombus formation is investigated in this study. Micro-machined molds were used to manufacture the chips by pouring PDMS in it. The PDMS was cured in an oven at 65 °C. Next, holes were made for later connection of the flow chamber to the tubing, and the PDMS was plasma bonded to a microscopic glass plate.

### *In vitro* experiment protocol

In Fig. 6c the set-up of the experiment is shown. The chip was coated overnight with 100 μg/mL type I collagen (Chronopar, Chronolog Corp., Havertown, USA) solution in 0.9% saline at room temperature. At the start of an experiment, the chip was positioned on the brightfield microscope (DM6000 B, Leica Microsystems, Wetzlar, Germany) table and was connected via tubing to a blood reservoir elevated by a tripod (inlet, open syringe) and to a petri dish that was placed on a mass balance (outlet). The elevated blood reservoir drove the flow through the channel. Blood was pipetted into the reservoir through 30 μm filter paper to remove small aggregates from the blood that can occlude the tubing or channel. The blood level in the reservoir was kept constant during the experiment. The blood level in the reservoir determines the hydrostatic pressure with which the experiment was driven. The experiment started when the blood reaches the start of the channel. During the experiment, an image was acquired every 500 ms with a high-resolution CCD camera (Pixelfly, PCO, Kellheim, Germany) using the open source software μManager^37^. The blood flowing out of the chip was measured by a mass balance (OHAUS SCOUT balance, Switserland) with an accuracy of 0.01 g. At the same time the evaporation of the blood-water mixture was measured with another mass balance and a petri dish with a similar blood-water mixture. The mass balance data was visualized and saved using LabView (National Instruments Corp., Austin, USA). The number of experiments performed with both devices perfused with PRP or WB at multiple pressure differences is given in Table 1. All experiments were performed at least twice per blood sample.

### Shear rates in the flow chamber of both microfluidic devices

The shear rates in the stenotic section were estimated by: ${\dot{\gamma }}=\frac{6Q}{wh^{2}}$ where *Q* denotes the volumetric flow obtained using the mass balance, *w* is the width of the channel and *h* is the height of the channel (see Table 1). Additionally, continuous and cell-based simulations were performed to obtain the shear rates and stress in the flow chambers for all experiments. The shear rate quantification results were presented in [Van Rooij et al., submitted for publication]. From this study was concluded that continuous blood flow simulations underestimated the shear rate by about 25% and overestimated the shear stress by a factor of two compared to cell-based blood flow simulations. In this study, the shear rate approximation and the continuum simulations were used to determine the shear rates in both microfluidic devices, because this was necessary to compare the results to the literature, as continuum simulations seems to be the standard in the literature^5,7,38,39^.

**Table 1:** Shear rates for whole blood and platelet-rich plasma estimated using the following relationship: ${\dot{\gamma }}=\frac{6Q}{wh^{2}}$. Additionally, the number of blood samples (animals) N that were used for each case and the average of the starting flow rates Q are given.

Device	WB or PRP	Δh (mm)	N (−)	Q (g·min^−1^)	${\dot{\gamma }}$ (s^−1^)
Van Rooij	WB	55	10	0.09	4,100
		115	10	0.15	7,100
		170	9	0.21	9,800
Van Rooij	PRP	55	6	0.12	7,100
		115	7	0.24	13,000
Casa	WB	70	7	0.13	3,900
		100	8	0.19	5,600
		160	8	0.36	11,000
Casa	PRP	70	5	0.31	9,100
		100	6	0.43	13,000

### Data and image processing

#### Lag and occlusion time

The mass balance data was collected using LabView at a frequency of 1 Hz. The data was filtered using a moving-average filter over a subset of six data points. The same was done for the evaporation data that was used to correct for changes in mass over time. To obtain the occlusion times, the maximum of the mass data without evaporation was taken, because this was the point at which the mass stayed constant in time in most cases, i.e. when the flow stopped. However, for a couple of cases the maximum consisted of multiple points, here the average time was chosen as occlusion time, because the evaporated corrected mass data showed no change in mass from that point. The flow rates were measured by taking the rate over a period of 1s of the measured mass data.

The lag time was calculated using the flow rate derived from the mass data as discussed above. The definition of the lag time by Li et al.^28^ was used in this study: 48% of the initial flow rate was estimated as the point at which the thrombus reached 48% of its volume, i.e. $Q(t_{\text {lag time}})=0.48 \cdot Q(t=0)$. Additionally, the height of the clot (y-direction) was estimated from the flow rate. The assumption of a constant pressure difference ($\frac{dp}{dx}$) in the channel was made and the following relation between flow rate *Q* and height *h* was used: $\frac{dp}{dx}=\frac{12Q\nu \rho }{wh^{3}}$ with the kinematic viscosity of blood *ν* (WB: 3.3·10^−6^ m^2^s^−1^, PRP: 1.1·10^−6^ m^2^s^−1^), the density of blood *ρ* (WB: 1,060 kg·m^−3^, PRP: 1,025 kg·m^−3^) and, the width of the channel *w* (see Supplementary Information Fig. S1 online).

The occlusion time and lag times were obtained for each individual experiment and averaged per blood sample. The number of blood samples (animals) is given in the graphs in the results section denoted by N. The average of these values per blood sample were defined as the occlusion time and lag time for each type of experiment. The standard errors of those averages were obtained and shown in the plots. Additionally, a two-tailed student’s t-test was performed with an α-level of 0.01 and significant differences were reported if *p* < 0.01. The t-value (t) and the degrees of freedom are given for each significant test.

#### Platelet aggregate growth and shrinkage

**Figure 7:**
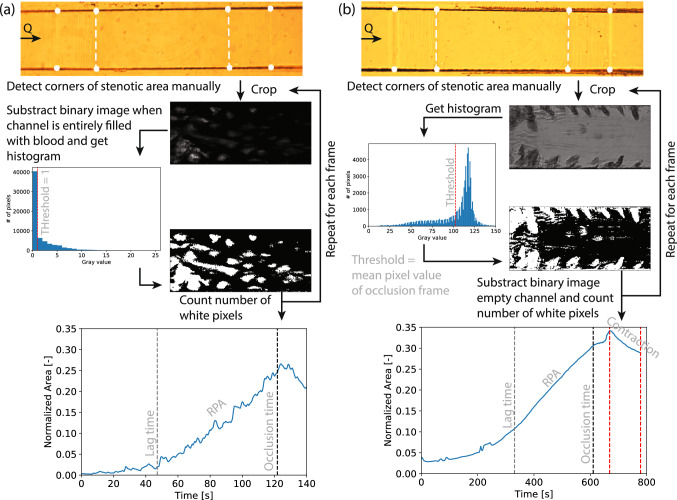
Schematic overview of image analysis for (**a**) WB experiments and (**b**) PRP experiments. In this overview Casa’s chip was used as an example. The area between the dashed white lines is the stenotic section and is used in the analysis. The flow direction is represented by the black arrow assigned with Q. The dashed gray line represents the lag time and the dark gray line represents the occlusion time in the normalized area graphs. The RPA rate is derived between the light gray and dark gray line and the shrinkage rate is defined between the two red lines shown in the normalized area graph of the PRP experiment.

The rapid platelet accumulation (blue) growth rate and shrinkage rate were analyzed using the microscopic images acquired during the experiments. The amount of area that has increased per unit time and the amount of area that has decreased (shrinkage) was called RPA rate and shrinkage rate, respectively. An overview of the analyzing steps is shown in Fig. 7 for both WB and PRP. The green channel of the images was used, because this channel gave the best contrast. This channel was converted to gray scale and cropped in a way that only the stenotic part of the channel was in the image, using the coordinates from the expansion and contraction detected manually. For the WB experiments, a gray scale image at the time that the channel was entirely filled with blood at the start of the experiment was subtracted from the gray scale image that was analyzed over time. The histogram of the occlusion frame gray scale image (for WB the image after subtraction) was used to calculate a cut-off pixel value that turns the gray scale image into a binary image. Those cut-off pixel values were different for PRP and WB, because for PRP a dark color represented the platelet aggregate; while for WB, platelet aggregates were represented with a bright color. This difference is caused by the presence of red blood cells that absorb the light, when there are less RBCs present in the flow chamber more light will go through to hit the sensor, this will happen when platelets start to attach to the collagen in the flow chamber. This cut-off pixel value for WB was one due to the subtraction, and for PRP, it was the averaged gray value (sum of all gray values/number of pixels). For the PRP experiments, the 10th frame of the binary image was subtracted from all binary images in time, such that the boundaries of the chip would be neglected in the pixel count. Next, the number of white pixels (value 1) was counted, because they represent the platelet aggregate. The area of the clot was then calculated using the scale of the images. To compare the growth rate and shrinkage rate among both chips, the normalized area was evaluated by dividing the area of the aggregate by the total area of the stenotic part. To get more insight into the growth of the aggregate in the y-direction, the height of the clot was derived from the flow rate as explained previously.

## Supplementary information

Supplementary Information 1

Supplementary Information 2

Supplementary Information 3

Supplementary Information 4

Supplementary Information 5
