# Supplementary material for: Biorheology of occlusive thrombi formation under high shear: in vitro growth and shrinkage
Source: Sci Rep. 2020 Oct 29;10:18604. doi: 10.1038/s41598-020-74518-7 (PMC7596481; doi:10.1038/s41598-020-74518-7)
Supplement: Supplementary file 1 — Supplementary Information 1 [file 41598_2020_74518_MOESM1_ESM.pdf]

Biorheology of occlusive thrombi formation under high shear: *in vitro* growth and shrinkage

Britt J.M. van Rooij[1], Gábor Závodszky[1], Alfons G. Hoekstra[*,1], and David N. Ku[2]

1 Computational Science, Institute for Informatics, University of Amsterdam, The Netherlands

2 GWW School of Mechanical Engineering, Georgia Institute of Technology, United States of America

* a.g.hoekstra@uva.nl

Mass data post-processing

An example of the post-processing and analysis of the mass data is performed is shown in Fig. S1.

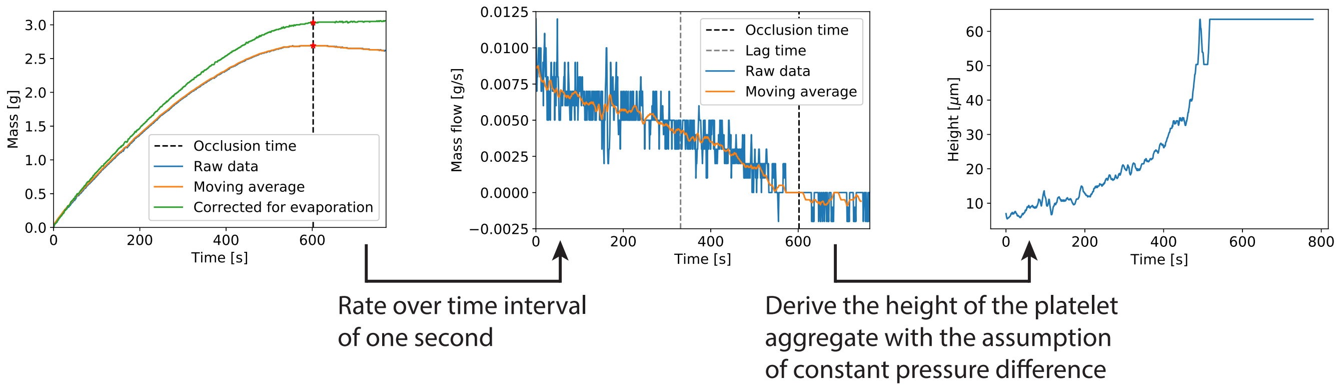

Figure S1: Schematic overview of mass balance post-processing to obtain the thrombus height.

Shear rate in the flow chambers

The shear rates and shear stress in both flow chambers at different pressure height were obtained using continuous blood flow simulations. The simulation results of Van Rooij's flow chamber are shown in Fig. S2. It can be seen that the shear rate is the highest in the stenotic part and in particular at the stenotic corners.

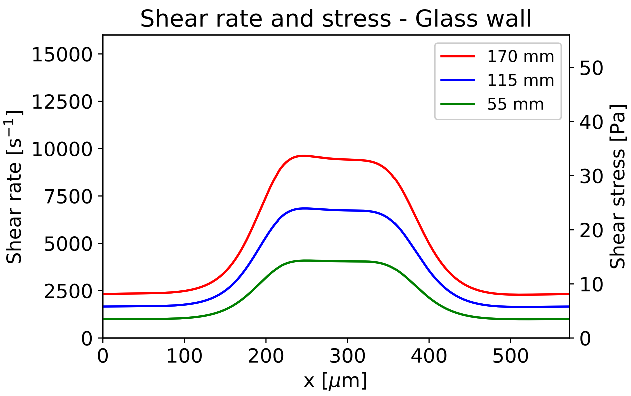

(a)

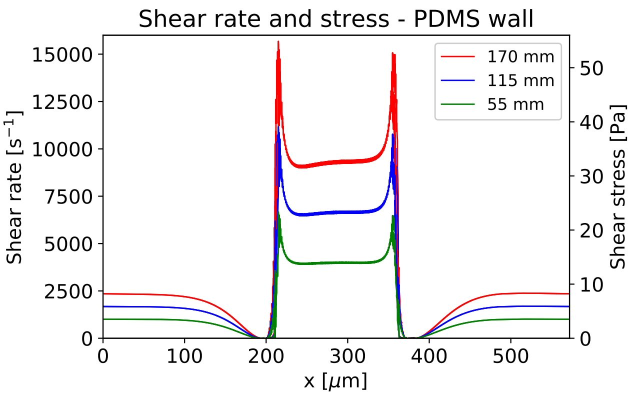

(b)

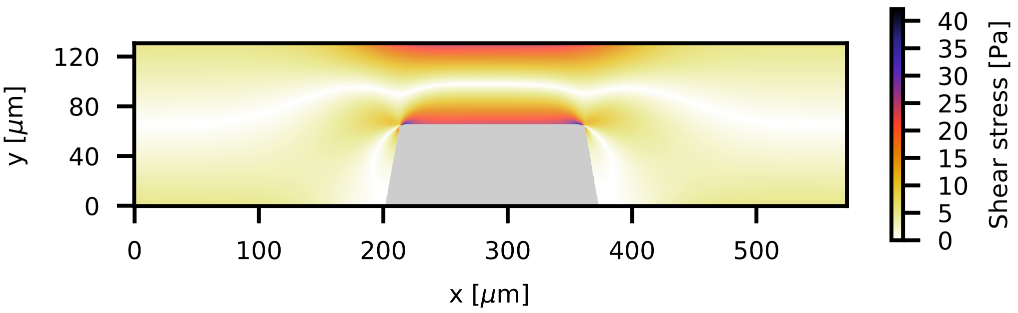

(c)

Figure S2: The shear rate and shear stress (a) along the x-axis at 1 μm from the top wall (y = 230 μm, non-stenotic) and (b) along the x-axis at 1 μm from the bottom wall (y = 0-65 μm, stenotic) of Van Rooij's flow chamber obtained in the WB continuous simulations. The shear rate and stress are given for the three different inlet pressure levels: 55 mm (green), 115 mm (blue) and 170 mm (red). (c) The shear stress averaged over the z-direction in Van Rooij's flow chamber at a height of 115 mm.

Residence time platelets

A cell-based simulation of the PRP experiments was performed with $\dot{\gamma} = 6,700$ s^{-1} and $\dot{\gamma} = 13,000$ s^{-1} in the stenotic section of Van Rooij's flow chamber. The simulated platelet-rich plasma contained 941 platelets and a viscosity of 1.1 m^{2}s^{-1} and a density of 1025 kg·m^{-3} were used. A time step of $5 \cdot 10^{-8}$ seconds and a lattice size of $5 \cdot 10^{-7}$ μm were used in the cell-based simulations. The residence times of the platelets in both simulations are shown in Figure S3. It is clearly visible that the residence times are higher for the low shear case (10-30 μs) in comparison to the high shear case (3-10 μs). Additionally, a plasma-free layer of about 10 μm in height is observed on both sides of the flow chamber. This might be caused by the lift force on the platelets due to the presence of the wall of the flow chamber.

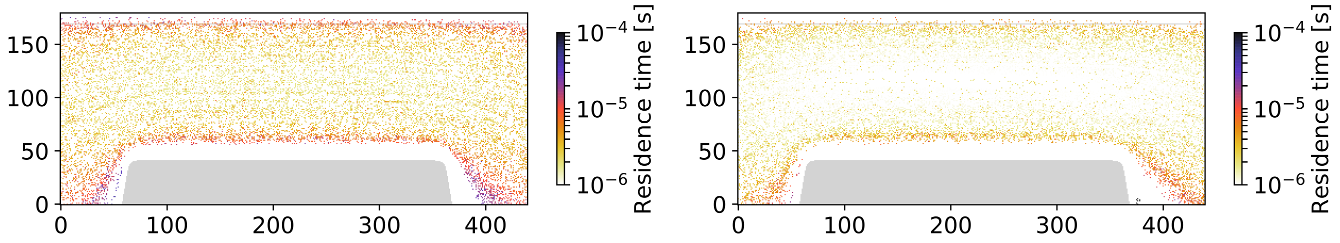

(a) (b)

Figure S3: The residence time of platelets in a cell-based simulation for (a) shear rate 6,700 s^{-1} and (b) shear rate 13,000 s^{-1} in the stenotic section of Van Rooij's flow chamber are shown. The residence time is defined as the time it takes for a platelet to translocate its own diameter (2 μm).

Supplementary videos

A video of each experiment is included as supplementary material. In Casa's flow chamber the dashed gray lines indicate the contraction and the expansion section of the chamber with in between them the stenotic section and in Van Rooij's flow chamber they indicate the start and the end of the stenotic section. The videos have been increased in speed by a factor of 5 for WB experiments and by a factor of 10 for PRP experiments.

- Supplementary video S1: WB experiment in Casa's flow chamber.
- Supplementary video S2: PRP experiment in Casa's flow chamber.
- Supplementary video S3: WB experiment in Van Rooij's flow chamber.
- Supplementary video S4: PRP experiment in Van Rooij's flow chamber.

Supplementary microscopic images

In Table S1 and S2 (brightfield) microscopic images at lag and occlusion time of an experiment are shown for both WB and PRP. For each PRP experiment, an image at the end of the experiment is given, because for PRP the shrinkage of the clot is measured en represented as well.

Table S1: Overview of WB and PRP experiments using Casa's flow chamber at lag and occlusion time. For PRP experiments an image at the end of the experiment is also shown. The blood was flowing from left to right.

	Lag time	Occlusion time	End of experiment
Casa - PRP (9,100 s^{-1})	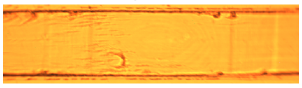	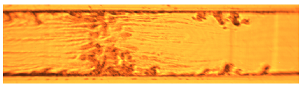	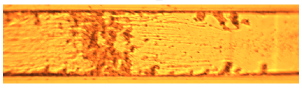
Casa - PRP (9,100 s^{-1})	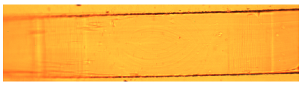	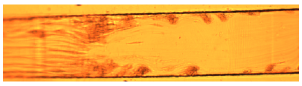	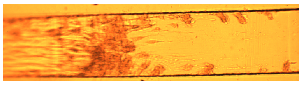
Casa - PRP (13,000 s^{-1})	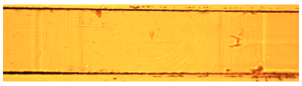	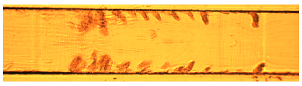	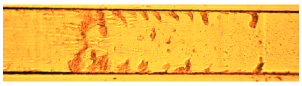
Casa - PRP (13,000 s^{-1})	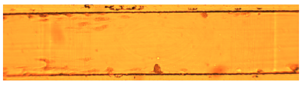	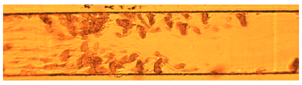	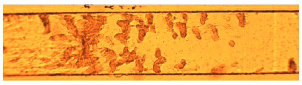
Casa - WB (3,900 s^{-1})	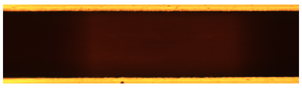	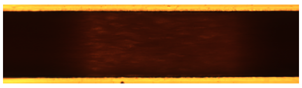	
Casa - WB (3,900 s^{-1})	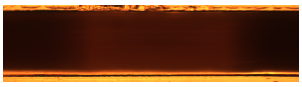	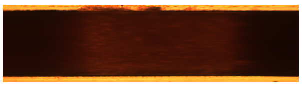	
Casa - WB (5,600 s^{-1})	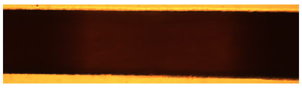	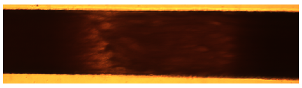	
Casa - WB (5,600 s^{-1})	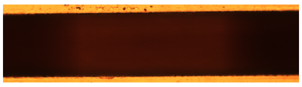	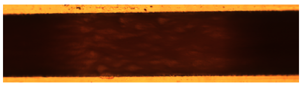	
Casa - WB (11,000 s^{-1})	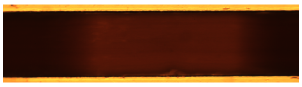	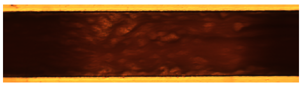	
Casa - WB (11,000 s^{-1})	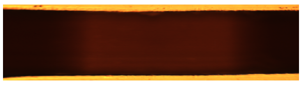	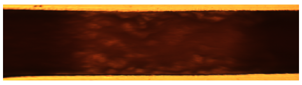	

Table S2: Overview of WB and PRP experiments using Van Rooij's flow chamber at lag and occlusion time. For PRP experiments an image at the end of the experiment is also shown. The blood was flowing from left to right.

	Lag time	Occlusion time	End of experiment
Van Rooij - PRP (7,100 s^{-1})	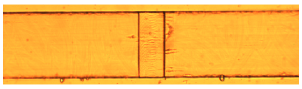	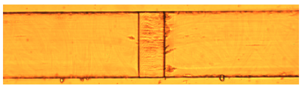	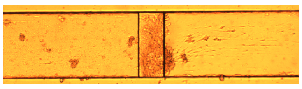
Van Rooij - PRP (7,100 s^{-1})	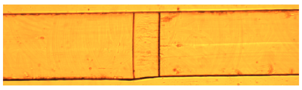	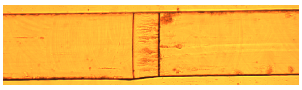	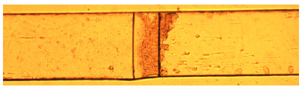
Van Rooij - PRP (13,000 s^{-1})	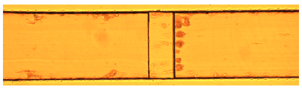	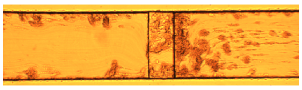	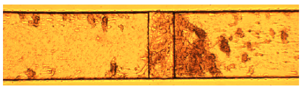
Van Rooij - PRP (13,000 s^{-1})	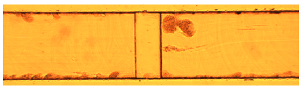	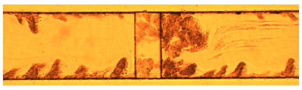	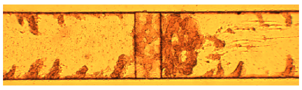
Van Rooij - WB (4,100 s^{-1})	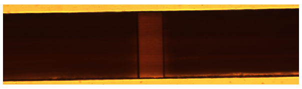	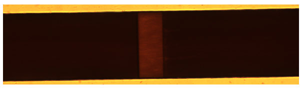	
Van Rooij - WB (4,100 s^{-1})	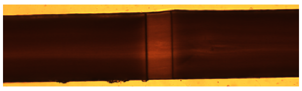	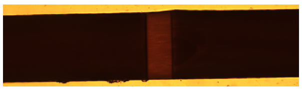	
Van Rooij - WB (7,100 s^{-1})	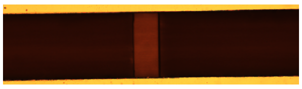	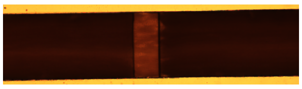	
Van Rooij - WB (7,100 s^{-1})	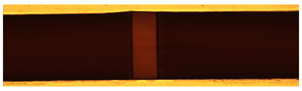	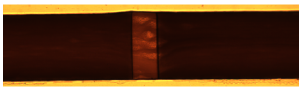	
Van Rooij - WB (9,800 s^{-1})	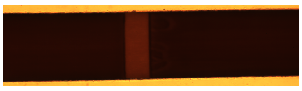	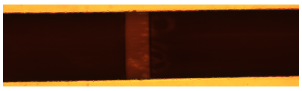	
Van Rooij - WB (9,800 s^{-1})	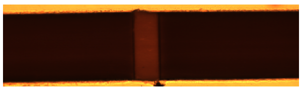	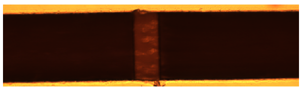	
